# Untargeted metabolomics reveal signatures of a healthy lifestyle

**DOI:** 10.1038/s41598-024-64561-z

**Published:** 2024-06-13

**Authors:** Wimal Pathmasiri, Blake R. Rushing, Susan McRitchie, Mansi Choudhari, Xiuxia Du, Alexsandr Smirnov, Matteo Pelleigrini, Michael J. Thompson, Camila A. Sakaguchi, David C. Nieman, Susan J. Sumner

**Affiliations:** 1 Department of Nutrition, University of North Carolina at Chapel Hill, Chapel Hill, NC 27599 USA; 2 Nutrition Research Institute, University of North Carolina at Chapel Hill, Kannapolis, NC 28081 USA; 3 College of Computing and Informatics, University of North Carolina at Charlotte, Kannapolis, NC 28081 USA; 4 Department of Molecular, Cell, and Developmental Biology, University of California Los Angeles, Los Angeles, CA USA; 5 Human Performance Laboratory, Department of Biology, Appalachian State University, North Carolina Research Campus, Kannapolis, NC 28081 USA

**Keywords:** Metabolomics, Mass spectrometry, Lifestyle, Physical activity, Diet, Obesity, Predictive markers, Biomarkers, Metabolomics

## Abstract

This cross-sectional study investigated differences in the plasma metabolome in two groups of adults that were of similar age but varied markedly in body composition and dietary and physical activity patterns. Study participants included 52 adults in the lifestyle group (LIFE) (28 males, 24 females) and 52 in the control group (CON) (27 males, 25 females). The results using an extensive untargeted ultra high-performance liquid chromatography-high resolution mass spectrometry (UHPLC-HRMS) metabolomics analysis with 10,535 metabolite peaks identified 486 important metabolites (variable influence on projections scores of VIP ≥ 1) and 16 significantly enriched metabolic pathways that differentiated LIFE and CON groups. A novel metabolite signature of positive lifestyle habits emerged from this analysis highlighted by lower plasma levels of numerous bile acids, an amino acid profile characterized by higher histidine and lower glutamic acid, glutamine, β-alanine, phenylalanine, tyrosine, and proline, an elevated vitamin D status, higher levels of beneficial fatty acids and gut microbiome catabolism metabolites from plant substrates, and reduced levels of N-glycan degradation metabolites and environmental contaminants. This study established that the plasma metabolome is strongly associated with body composition and lifestyle habits. The robust lifestyle metabolite signature identified in this study is consistent with an improved life expectancy and a reduced risk for chronic disease.

## Introduction

Advances in mass spectrometry and data computing capabilities have expanded the use of metabolomics in health- and disease-related research projects. Targeted and untargeted metabolomics can be used in the large-scale study of the metabolome, defined as the collection of small molecules and their interactions, within a biological system. Targeted metabolomics typically involves a priori selection of metabolites and pathways for testing a hypothesis. Using untargeted methods, over 20,000 metabolites have been detected in humans and thousands of metabolites can be measured simultaneously in plasma, serum, urine, sweat, fecal, and other types of samples^1^. Metabolomics profiling has advanced scientific understanding of underlying metabolic pathways in health promotion and disease prevention, expanded appreciation for metabolic heterogeneity, and identified new biomarkers to use in tracking responses to medical treatments and lifestyle changes^2^.

Numerous studies indicate that adherence to recommended lifestyle habits such as participation in regular physical activity, consumption of high-quality diets, maintenance of healthy body weight, moderation of alcohol intake, and avoidance of tobacco use is associated with increased life expectancy and reduced risk of most chronic diseases^3–5^. The principal mechanisms explaining these relationships and the wide-ranging human responses are still poorly understood. Investigations have attempted to define unique metabolite signatures related to cardiorespiratory fitness^6–10^, healthy dietary patterns^11–16^, the aging process^17^, and obesity^18–23^, but disparate study designs and methodologies and the emergent nature of this field of study have forestalled scientific consensus. Several studies have investigated the unique metabolomic profile associated with two or more combined healthy lifestyle behaviors^24–27^. Kaspy et al.^26^ systematically reviewed nine studies and concluded that limited evidence supported positive metabolite shifts with combined healthy lifestyle behaviors including elevations in phosphocreatine and beneficial fatty acids and lower acylcarnitines and trimethylamine N-oxide (TMAO). Excessive adiposity that typically develops from an energy imbalance due to physical inactivity and overeating has been linked to a strong metabolite signature including elevations in the nucleotide urate, amino acids such as glutamate, kynurenate, alanine, tyrosine, and branched chain amino acids (BCAAs), lipids such as acylcarnitines, sphingomyelin, carnitine, some types of bile acids, and triglycerides, and carbohydrates such as glucose and mannose. Lower concentrations of some lipids such as cortisone, phospholipids and lysolipids, and high-density lipoprotein (HDL)-related fats, and xenobiotics such as cinnamoylglycine have also been related to obesity^18–23^.

Metabolomics is a systems biology approach ideally suited to revealing the complex interactions between lifestyle habits and underlying physiological mechanisms^24–29^. The aim of this study was to investigate differences in the plasma metabolome in two groups of adults that were distinctly different in body composition and dietary and physical activity patterns. Of the various multi-component lifestyle studies reviewed by Kaspy et al.^26^, none used a cross-sectional design comparing widely disparate groups of adults. The studies reviewed by Kaspy et al.^26^ included three randomized controlled trials with obese adults, three nested case–control studies (two with cancer patients), and three single-arm trials (two with obese children). The authors of this systematic review were limited in their ability to provide definitive conclusions on lifestyle-related metabolite shifts due to the heterogeneity among the study populations, and differences in study designs and metabolomic approaches used for biomarker detection^26^. This cross-sectional study of two groups of male and female adults varying widely in adherence to lifestyle recommendations used untargeted UHPLC-HRMS with more than 10,000 signals to provide an in-depth analysis of the associated metabolite signature. Cross-sectional studies allow widely disparate groups to be compared providing important data that can be followed up in subsequent epidemiological cohort and randomized clinical trials. The range of analytes detected on the UHPLC-HRMS system included those related to host metabolism, the environment, medication use, and food and microbial metabolites.

## Methods

### Study participants

The proteomics data and information regarding the study participants and methods from this cross-sectional study have been published in a separate paper^30^. The reader is referred to this paper for complete details and a brief description will be provided in this paper. Male and female study participants ages 25–75 years were recruited and voluntarily signed the informed consent. Procedures were approved by the Appalachian State University Human Subjects Institutional Review Board (IRB), Federal Wide Assurance (FWA) number: FWA00027456. Notice of IRB approval by expedited review was granted by the IRB (#21-0054) on 10/16/2020^30^. The research was performed in accordance with relevant guidelines and regulations, and informed consent was obtained from all study participants^30^.

Participants were not included if they were currently being treated for heart disease or cancer (excluding skin cancer), or medically complicated conditions (i.e., diabetes requiring insulin, uncontrolled high blood pressure). No restrictions were placed on diet, supplement usage, or medications^30^. Other inclusion criteria for the lifestyle group were similar to those used in previous lifestyle-metabolomics studies^26^: (1) Healthy, with no current history of chronic or infectious disease; (2) Not overweight or obese (body mass index less than 25 kg/m^2^); (3) High physical activity level (> 300 min per week, vigorous exercise); (4) Non-smoker for at least the previous three years; (5) Healthy dietary pattern (recommended intake of fruits, vegetables, whole grains, low-fat dairy products, healthy protein foods, and a low intake of salt, sugar, fats, and alcohol). Other inclusion criteria for the control group were as follows: (1) Healthy, with no current history of chronic or infectious disease; (2) Obese (body mass index greater than or equal to 30 kg/m^2^); (3) Sedentary or low physical activity level (< 150 min per week); (4) Unhealthy dietary pattern^30^.

### Study design and methods

This study employed a cross-sectional design that compared metabolite profiles in adults adhering (n = 52) or not adhering (n = 52) to lifestyle recommendations. Questionnaires provided information about their lifestyle and physical activity patterns, mood states, estimated VO2max, nutrient intake, and medical history^30^.

Participants reported to the lab at the scheduled appointment time in an overnight fasted state (i.e., no food, supplements, or beverages other than water for at least the previous 8 h). After 10–15 min of seated rest, resting heart rate (RHR) and blood pressure were measured. A 35 ml blood sample was collected from an arm vein. Venous blood samples were collected in ethylenediaminetetraacetic acid (EDTA) containing blood collection tubes. Plasma aliquots were prepared from EDTA containing blood collection tubes and stored in a − 80 °C freezer until analysis for metabolomics. Participants were taken into the performance lab for measurements of height, weight, waist circumference, sagittal abdominal diameter, leg/back and hand grip dynamometer strength, and body fat (bioelectrical impedance or BIA)^30^.

#### Untargeted metabolomics data capture and preprocessing

Details of the sample preparation, data acquisition, data preprocessing and metabolite identification and annotation^31–33^ are provided in the Supplementary Methods Material. Briefly, untargeted metabolomics data of randomized plasma samples (interspersed with 10% blanks, quality control study pools (QCSP), and NIST SRM 1950 plasma reference material) was acquired in positive mode on a Vanquish UHPLC system coupled with a Q Exactive™ HF-X Hybrid Quadrupole-Orbitrap™ Mass Spectrometer (UHPLC-HRMS; Thermo Fisher Scientific, San Jose, CA). Raw files for all study samples, QCSP, blank, and NIST reference material runs were uploaded to Progenesis QI (Waters Corporation, Milford, MA) for alignment and peak picking. Data was normalized^34,35^ to a reference QCSP sample using the “normalize to all” function in Progenesis QI^36^. Peaks detected by UHPLC-HRMS were identified or annotated using ADAP-KDB software^37^. The evidence basis for metabolite identifications and annotations^31–33^ to the in-house physical standards library (Ontology Level, OL), or Public Databases (PD), are described in the Supplementary Methods Material. It should be noted that metabolomics platforms cannot always distinguish between isomers and that multiple peaks may match the same compound. Additionally, one metabolic peak may match to multiple metabolites due to adduct formation or isobaric compounds.

#### Multivariate data analysis, and univariate statistics

Multivariate analysis was performed for the normalized UHPLC-HRMS data, using SIMCA 17.0 to reduce the dimensionality and to enable the visualization of the differentiation of the phenotypic groups (SIMCA 17, Sartorius Stedim Data Analytics, AB, Umeå, Sweden)^38,39^. Unsupervised multivariate analysis models were created using principal component analysis (PCA) and the scores plots were inspected to ensure that the QCSP samples were tightly clustered, and in the center of the study samples from which they were derived—a quality control method that is widely used in metabolomic studies^40^. Orthogonal partial least squares discriminate analysis (OPLS-DA) was used to determine the variable influence on projection (VIP), for the preprocessed UHPLC-HRMS data, to define the signals deemed important for differentiating the phenotypic groups. VIP ≥ 1.0 with a jack-knife confidence interval that did not include 0 were selected as important. The VIP statistic summarizes the importance of the signal in differentiating the phenotypic groups^39^. All models used a sevenfold cross-validation to assess the predictive ability (Q2) of the model. Additional statistical analyses were conducted using SAS 9.4 (SAS Institute Inc., Cary, NC), and included using a two-sided t-test with the Satterthwaite correction for unequal variances. Nominal p-values are reported for the comparison between lifestyle and the controls because this exploratory analysis was not powered for a specific hypothesis^41–43^. Metabolite peaks that met VIP ≥ 1 or p < 0.10 fold group differences ≥ │2.0│ were reported for the differentiation of the phenotypic groups in the metabolomics analysis. This discovery study did not use FDR correction because the study was not powered for a specific hypothesis^41–43^. This study determined linear combinations of metabolites that have variable importance to projection scores ≥ 1 that are important for determining differences between the lifestyle and control groups. P-values were reported in all cases. While some of these p-values may not be significant after FDR correction, these metabolites are still important to the signature that differentiates the lifestyle and control groups.

#### Lasso modeling

Lasso regression was used to consider another statistical model that reduces errors caused by overfitting. Group (LIFE, CON) discriminators based on the entire metabolomics dataset were constructed using penalized logistic regression. This analysis was conducted using the R packages “glmnet” (cran.r-project.org/web/packages/glmnet/)^44,45^ with the alpha parameter set to 1.0. This is equivalent to “Lasso” regression wherein the number of predictor variables in the categorization model is minimized. The normalized intensities for all metabolite peaks across 104 subjects were input into the algorithm with group status (LIFE, CON) as the binary category to be predicted. As it is well known that even penalized regression techniques will over-fit the training data when the number of predictor variables is much larger than the number of samples, a leave-one-out (LOO) protocol was used to get an estimate of how well a discriminator trained on this data might perform on new samples. The LOO approach consists of iterating over all N samples in the dataset. At each step one of the samples is withheld while a model is optimized over the other N-1 samples and a prediction is made for the sample that was held out. We computed receiver-operating characteristic (ROC) curves from the LIFE and CON group predictions obtained with a simple LASSO regression model. To compare ROC curves, we computed the area under the curve (AUC) and a p-value for obtaining such an AUC at random. These calculations were performed using the R package “pROC” (https://cran.r-project.org/web/packages/pROC/index.html).

#### Pathway enrichment and biological interpretation

Pathway enrichment analysis was conducted using the Mummichog algorithm^46^ in Functional Analysis module in Metaboanalyst 5.0^47–49^. The 10,535 features (m/z) that remained after data preprocessing were entered together with the mass-to-charge ratio (m/z), retention time, positive mode, the p-value, and fold change between the comparison of lifestyle versus controls for all subjects. A p-value cut-off of 0.05 was used for the size of the permutation group that the algorithm used for selecting significant features for metabolite matching. A 3 ppm mass tolerance was used for mass accuracy for annotating peaks to metabolites and identifying candidate pathways. All possible metabolites that were matched by m/z were searched in the Homo sapiens (human) [MFN] pathway library. The experimental list of metabolites was compared to a null distribution of randomly generated m/z features from the reference library to determine pathway significance^46^. Significance was reported as uncorrected p-values. In addition to the pathway analysis using MetaboAnalyst, biochemical pathway interpretation was conducted with a classical approach of assessing the connection between analytes that met the criteria for being most important (VIP ≥ 1 and p < 0.05 for group fold differences ≥ │1.8│) between LIFE and CON groups. Some metabolites are represented in more than one metabolic pathway.

## Results

### Subject characteristics

This study employed a cross-sectional design and compared 52 subjects in the lifestyle group (LIFE) and 52 in the control group (CON) (Table 1). The sex distribution was comparable between groups (LIFE, 28 males, 24 females) and CON ^27males,25females^ (Χ^2^ = 0.039, p = 0.844). Analyses were conducted for all study participants combined. Age, education level, and height did not differ significantly between LIFE and CON groups (Table 1). Several measures of body composition differed between LIFE and CON groups (Table 1). These included the body mass index (BMI), fat mass index (FMI), body fat percentage, and sagittal abdominal diameter (SAD). Estimated aerobic capacity (VO2max) and total physical activity calculated as MET-min/week differed between the LIFE and CON groups (p < 0.001) (Table 1). Fruit and vegetable intake was higher and red meat intake lower in LIFE vs. CON (both p < 0.001). Strength was measured using leg/back and handgrip dynamometers and differed between groups after adjustment for body mass (p < 0.001) (Table 2). Diastolic blood pressure and the resting heart rate were significantly lower in LIFE vs. CON (p < 0.001) (Table 1).

**Table 1:** Subject characteristics.

Variable	Lifestyle (n = 52) (28M, 24F)	Control (n = 52) (27M, 25F)
Age (yrs)	47.5 ± 12.2	51.1 ± 10.5
Education (yrs)	16.3 ± 3.4	15.2 ± 3.1
Weight (kg)	67.8 ± 11.8*	101.5 ± 15.5
Height (cm)	172 ± 9.0	171 ± 9.5
Waist circumference (cm)	82.5 ± 7.5*	112.2 ± 11.5
Sagittal abdominal diameter (SAD) (cm)	17.5 ± 2.13*	26.9 ± 3.4
BMI (body mass kg/height m^2^)	22.9 ± 2.6*	34.5 ± 4.0
Body fat (%)	22.6 ± 7.3*	40.8 ± 7.2
Fat mass index (fat mass kg/height m^2^)	5.32 ± 1.90*	14.2 ± 3.57
VO_2max_ (ml kg^−1^ min^−1^)	37.7 ± 8.8*	20.3 ± 8.4
Physical activity (MET-min/week)	5463 ± 3723*	2319 ± 2614
Systolic blood pressure (sBP) (mm Hg)	115 ± 14.5	117 ± 14.5
Diastolic blood pressure (dBP) (mm Hg)	67.8 ± 10.0*	73.0 ± 10.5
Fruit and vegetable (servings/day)	5.1 ± 2.0*	2.9 ± 1.4
Red meat (servings/day)	0.45 ± 0.64*	1.21 ± 0.72

Data expressed as mean ± standard deviation. *p < 0.001, group difference.

**Table 2:** Library matched metabolite peaks that most significantly differentiated LIFE (n = 52) and CON (n = 52) groups (VIP ≥ 1 and p < 0.05 for group fold differences ≥ │1.8│).

Metabolite peak^a^	Ontology level (OL)	Metabolite	VIP	p-value^b^	Fold difference^c^
0.63_148.0602m/z	OL_1	3-Methylaspartate	2.6	1.30E−09	− 2.5
	OL_1	Glutamic acid	2.6	1.30E−09	− 2.5
	OL_1	N-Acetylserine	2.6	1.30E−09	− 2.5
	OL_2a	N-Methyl-d-aspartic acid	2.6	1.30E−09	− 2.5
	OL_2a	O-Acetylserine	2.6	1.30E−09	− 2.5
12.14_330.2553n	OL_2b	Docosapentaenoic acid	2.4	2.55E−07	− 6.1
13.06_373.2733m/z	OL_1	α-Muricholic acid	2.4	2.61E−07	− 2.6
	OL_1	β-Muricholic acid	2.4	2.61E−07	− 2.6
	OL_2b	γ-Muricholic acid	2.4	2.61E−07	− 2.6
	OL_2b	7-Ketochenodeoxycholate	2.4	2.61E−07	− 2.6
	OL_2b	Allocholic acid	2.4	2.61E−07	− 2.6
	OL_2b	Cholic acid	2.4	2.61E−07	− 2.6
	OL_2b	Ursocholic acid	2.4	2.61E−07	− 2.6
1.01_129.0787n	OL_1	Pipecolic acid	2.2	2.14E−04	1.8
16.63_437.3015m/z	OL_2a	Diosgenin	2.1	2.09E−05	1.9
0.68_309.1050n	OL_2b	N-Acetylneuraminic acid	2.1	2.41E−06	− 2.0
17.26_568.4279n	OL_2a	Lutein	2.0	1.41E−05	2.9
11.74_430.2950m/z	OL_2b	Glycylcholic acid	2.0	2.35E−05	− 3.2
0.74_308.0903m/z	OL_1	Reduced glutathione	1.9	3.66E−05	2.3
13.46_243.2100m/z	OL_2b	γ-Linolenic acid	1.9	5.88E−05	5.9
11.22_347.2213m/z	OL_1	Cortexolone	1.9	4.56E−05	− 1.9
	OL_1	Corticosterone	1.9	4.56E−05	− 1.9
9.98_288.1587m/z	OL_2b	Dihydromorphine	1.8	1.63E−04	− 4.1
4.31_134.0599m/z	OL_2a	Acetaminophen	1.7	1.70E−02	− 1.9
16.16_432.3234n	OL_2b	1,24,25-Trihydroxyvitamin D3	1.7	2.58E−03	− 1.8
4.70_144.0806m/z	OL_1	Tryptamine	1.7	7.73E−04	2.0
	OL_2b	3-(2-Hydroxyethyl)indole	1.7	7.73E−04	2.0
15.68_430.3081n	OL_2b	7α-hydroxy-3-oxo-4-cholestenoic acid	1.7	8.45E−04	6.7
5.31_228.0625m/z	OL_2b	Indolelactic acid	1.6	1.78E−03	− 2.7
	OL_2b	trans-Cinnamoylglycine	1.6	1.78E−03	− 2.7
10.85_180.1015m/z	OL_2a	Propham	1.6	1.15E−03	− 4.4
	OL_2b	Salsolinol	1.6	1.15E−03	− 4.4
0.58_188.1520n	OL_1	N,N,N-Trimethyllysine	1.6	2.57E−04	− 1.9
	OL_2b	Propamocarb free base	1.6	2.57E−04	− 1.9
1.48_190.0704m/z	OL_1	N-Acetylglutamate	1.6	5.15E−03	− 1.8
	OL_2a	3-Dehydroshikimic acid	1.6	5.15E−03	− 1.8
4.83_268.1511m/z	OL_1	2-Methylbutyroylcarnitine	1.6	1.01E−02	− 1.8
	OL_1	Isovaleryl-l-carnitine	1.6	1.01E−02	− 1.8
	OL_1	Valeryl-l-carnitine	1.6	1.01E−02	− 1.8
7.79_206.0809m/z	OL_1	trans-Cinnamoylglycine	1.5	9.80E−04	2.5
	OL_2a	Dioxacarb	1.5	9.80E−04	2.5
16.78_328.2392n	OL_2b	Docosahexaenoic acid	1.5	2.52E−03	2.1
7.79_148.0523n	OL_2b	trans-Cinnamic acid	1.5	1.93E−03	2.6
14.13_392.2921n	OL_2b	Sodium deoxycholate	1.5	1.20E−03	− 1.8
	OL_2b	Chenodeoxycholic acid	1.5	1.20E−03	− 1.8
	OL_2b	Murideoxycholic acid	1.5	1.20E−03	− 1.8
0.96_117.0428n	OL_1	N-Acetylglycine	1.5	5.17E−04	1.8
0.83_277.1025m/z	OL_2a	Pseudouridine	1.5	8.86E−04	− 1.8
1.01_82.0651m/z	OL_2a	5-Aminopentanoate	1.4	8.73E−03	2.0
	OL_2a	Betaine	1.4	8.73E−03	2.0
	OL_2a	Nicotine	1.4	8.73E−03	2.0
9.25_150.0677n	OL_1	Hydrocinnamic acid	1.4	7.82E−03	1.9
1.01_160.0602m/z	OL_2a	Glucosaminate	1.4	4.96E−03	2.6
	OL_2a	1-Methylnicotinamide	1.4	4.96E−03	2.6
11.48_266.1382m/z	OL_2a	Monocyclohexyl phthalate	1.4	1.54E−03	− 2.5
5.82_170.0210m/z	OL_2a	trans-4-Hydroxy-l-proline	1.6	1.71E−03	− 1.8
1.53_248.1484m/z	OL_1	Hydroxybutyrylcarnitine	1.4	6.57E−03	− 2.4
	OL_2b	Prometon	1.4	6.57E−03	− 2.4
11.37_334.2139n	OL_2b	Prostaglandin B2	1.4	5.06E−03	4.1
13.46_392.2921n	OL_1	Ursodeoxycholate	1.4	2.74E−03	− 2.3
	OL_1	Murideoxycholic acid	1.4	2.74E−03	− 2.3
	OL_2b	Deoxycholic acid	1.4	2.74E−03	− 2.3
	OL_2b	Chenodeoxycholic acid	1.4	2.74E−03	− 2.3
13.46_339.2677m/z	OL_2b	Dehydrolithocholic acid	1.4	2.92E−03	− 2.8
8.72_416.3156m/z	OL_2b	Glycolithocholic acid	1.4	3.62E−03	− 2.0
5.31_179.0581n	OL_1	Hippuric acid	1.4	5.55E−03	1.8
14.16_401.2658m/z	OL_1	Nordeoxycholic acid	1.3	6.63E−03	− 1.8
7.86_260.0887m/z	OL_2a	3-Hydroxycarbofuran	1.3	4.78E−03	− 2.1
0.74_365.1050m/z	OL_1	Lactose	1.3	6.49E−03	− 1.9
	OL_1	Sucrose	1.3	6.49E−03	− 1.9
	OL_1	Trehalose dihydrate	1.3	6.49E−03	− 1.9
	OL_2a	Palatinose	1.3	6.49E−03	− 1.9
17.15_316.2514m/z	OL_2a	Adrenic acid	1.3	9.31E−03	3.5
14.19_252.6229m/z	OL_2a	N-Acetyl leukotriene E4	1.3	3.34E−02	− 2.2
16.85_401.3408m/z	OL_1	7-Ketocholesterol	1.3	8.46E−03	− 2.2
	OL_1	7α-Hydroxy-4-cholesten-3-one	1.3	8.46E−03	− 2.2
11.03_357.2032m/z	OL_2b	Prostaglandin B2	1.3	8.85E−03	2.3
0.74_216.1226m/z	OL_2a	Epinephrine	1.3	4.23E−02	1.9
7.00_219.0624m/z	OL_2b	3,4-Dimethoxyphenylacetic acid	1.2	1.66E−02	3.8
8.55_233.0778m/z	OL_1	3,4-Dimethoxyphenylpropanoic acid	1.2	1.85E−02	2.1
10.79_375.2138m/z	OL_2b	Prostaglandin E2	1.2	2.40E−02	2.1
2.45_160.0756m/z	OL_2b	Indoleacetaldehyde	1.2	2.87E−02	2.1
	OL_1	Serotonin	1.2	2.87E−02	2.1
7.33_173.1048n	OL_1	Hexanoyl glycine	1.2	6.64E−03	2.8
	OL_2b	N-Acetylleucine	1.2	6.64E−03	2.8
9.57_189.0784n	OL_2b	IndolE−3-propionic acid	1.2	1.14E−02	− 2.3
	OL_2a	IndolE−3-methyl acetate	1.2	1.14E−02	− 2.3
15.73_433.3307m/z	OL_2a	Coprocholic acid	1.2	1.15E−02	− 2.2
8.55_151.0749m/z	OL_2a	(3,4-Dimethoxyphenyl)methanol	1.2	1.96E−02	2.0
	OL_2b	2-Phenylpropionate	1.2	1.96E−02	2.0
	OL_2b	3-Phenylpropanoic acid	1.2	1.96E−02	2.0
1.01_147.0649m/z	OL_2a	Fucose	1.2	2.52E−02	− 3.0
	OL_2a	Galactitol	1.2	2.52E−02	− 3.0
	OL_2a	Sorbitol	1.2	2.52E−02	− 3.0
2.45_160.0756m/z	OL_2a	Cotinine	1.2	2.87E−02	2.1
1.01_199.0211m/z	OL_1	Glucuronic acid-lactone	1.2	8.76E−03	− 3.1
	OL_1	Ascorbic acid	1.2	8.76E−03	− 3.1
4.74_134.0598m/z	OL_2b	Norepinephrine	1.1	2.38E−02	− 2.3
12.17_331.1146m/z	OL_2b	Mono (5-carboxy-2-ethylpentyl) phthalate	1.1	1.58E−02	− 4.4
7.55_183.0647m/z	OL_2a	3,4-Dimethoxybenzoic acid	1.2	6.37E−02	1.9
	OL_2a	2,4-Dimethoxybenzoic acid	1.2	6.37E−02	1.9
	OL_2b	1,3-Dimethyluric acid	1.2	7.63E−02	− 1.8
5.56_146.0689n	OL_2b	Glutamine	1.1	1.12E−02	− 1.8
10.46_301.1314n	OL_2b	10-Hydroxymorphine	1.1	3.09E−02	− 2.0
	OL_2b	Fenoxycarb	1.1	3.09E−02	− 2.0
	OL_2b	Furalaxyl	1.1	3.09E−02	− 2.0
	OL_2b	Oxymorphone	1.1	3.09E−02	− 2.0
	OL_2b	MorphinE-N-oxide	1.1	3.09E−02	− 2.0
16.56_285.2204m/z	OL_1	13-cis-retinal	1.1	2.33E−02	2.7
	OL_2b	Eicosapentaenoic acid	1.1	2.33E−02	2.7
0.74_189.1229m/z	OL_1	N-α-Acetyllysine	1.1	3.84E−02	− 1.9
13.72_495.3445m/z	OL_2a	2,6-di-tert-butyl-4-hydroxy-4-methylcyclohexa-2,5-dien-1-one	1.1	7.11E−02	4.4
9.79_183.0800m/z	OL_2b	9-Hydroxyfluorene	1.0	3.47E−02	− 2.2
5.31_175.0363m/z	OL_2a	4-hydroxyphenylacetic acid	1.0	3.44E−02	− 4.2
1.07_138.0524m/z	OL_2a	Proline	1.0	3.51E−02	− 3.7
5.56_491.1998m/z	OL_2a	Folinic acid	1.0	3.08E−02	− 2.0

The table is sorted by Variable Influence on Projection (VIP). See Supplementary Table S4 for additional important metabolite peaks with statistics.

^a^Mass spectrometry metabolomics platforms cannot always distinguish between isomers, and multiple peaks may match the same compound. Additionally, one peak may match multiple metabolites due to adduct formation or isobaric compounds. For the complete list of metabolites annotations or identifications of metabolite peaks see Supplementary Tables S1 and S2.

^b^t-test with Satterthwaite correction for unequal variances.

^c^Positive fold difference indicates that the mean value for the LIFE group was greater than for the CON group.

### Metabolomics comparisons and peak identification and annotation

Untargeted UHPLC-HRMS analysis was conducted on the preprocessed and normalized data with the 10,535 metabolite peaks that remained after peak filtration. The initial scores plot of the PCA showed tight clustering of QCSP and NIST reference plasma samples (Supplementary Methods Material with Fig. S1). All metabolite peaks in the preprocessed dataset were used for metabolite identification and annotation using ADAP-KDB software and both experimental standards library and public databases. A total of 1539 metabolites were identified or annotated using the in-house physical standards library. An ontology level OL_1 (RT, exact mass, and MS/MS) was reported for 418 metabolite matches, an OL_2a (RT, exact mass) for 527 metabolite matches, and OL_2b (exact mass, MS/MS) for 594 matches using the in-house experimental standards library (Supplementary Material, Table S1). A total of 122,527 metabolite annotations were made by library matching signals to public databases by exact mass and experimental MS/MS (PDa; 7567 annotations), exact mass and isotopic pattern (102,482 annotations, PDc), and exact mass (12,478 annotations, PDd) (Supplementary Material, Table S2). Signals that did not match the in-house library or public databases were labeled as unknown metabolite peaks. Results of this untargeted metabolomics study matched signals to isobaric and isomeric compounds, and future studies would be needed to resolve multiple peak matches.

### Multivariate and univariate statistics

**Figure 1:**
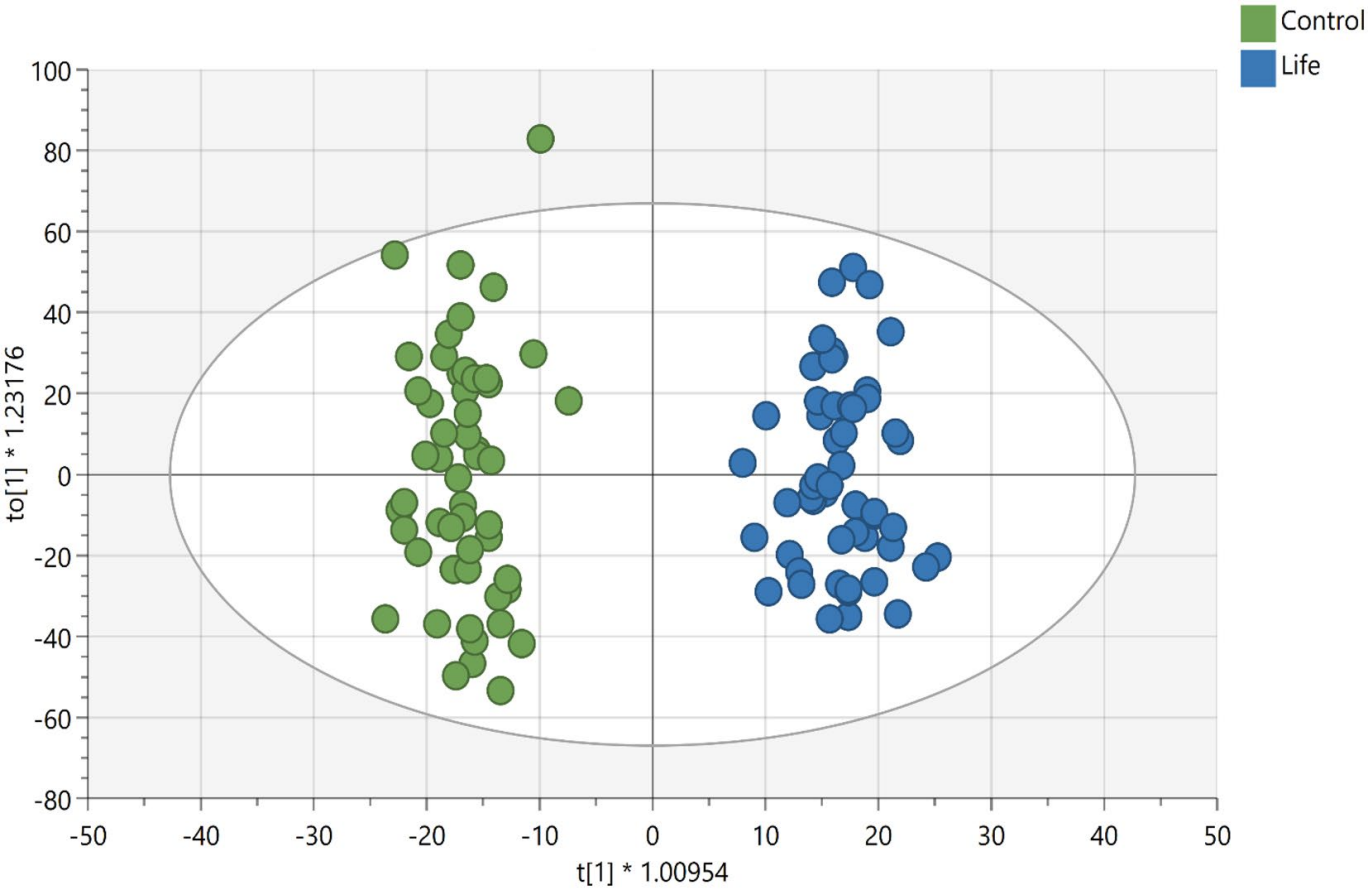
Scores plots of OPLS-DA of the metabolomics data obtained by UHPLC-HRMS analysis of plasma samples showing the differentiation of Lifestyle (LIFE) (blue, right-hand side) from Controls (CON) (green, left-hand side), R2X = 0.175; R2Y = 0.959); Q2 = 0.523, sevenfold cross validation).

The supervised OPLS-DA for plasma samples from the LIFE and CON groups (Fig. 1) showed strong model statistics for outcome (R2Y = 0.959) and prediction (Q2 = 0.523, sevenfold cross validation). Over 5300 signals met the criteria of VIP ≥ 1 or p < 0.10 or a fold change ≥ │2│ (Supplementary Material, Table S3). Over 3200 signals had p < 0.10, and over 2400 signals had p < 0.05 for comparison between LIFE and CON. Over 1200 signals had p < 0.01 for comparisons between LIFE and CON groups. A total of 486 important metabolite peaks (VIP ≥ 1) were matched to metabolites in the in-house physical standards library using ADAP-KDB software (Supplementary Material, Table S4). The most important metabolite peaks library matched to metabolites (VIP ≥ 1 and p ≤ 0.05 or fold group differences ≥ │1.8│) are shown in the Table 2.

### Lasso modeling

**Figure 2:**
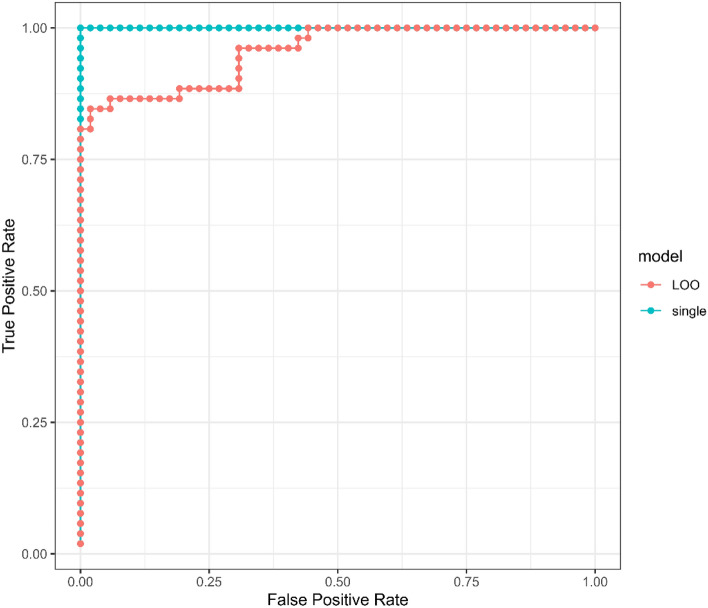
Receiver-operator-characteristic (ROC) curves for LIFE and CON group discriminators trained on the entire metabolomics dataset. The blue curve is derived from category scores obtained from a single model optimized on all 104 samples, while the red curve depicts category scores obtained for each of 104 samples using 104 separate models optimized on 103 samples (leave-one-out cross-validation, LOO-CV). The area under the curves (AUC) for the single model and LOO models were 1.0 and 0.96, respectively, with p values of 7.7e−19 and 5.6e−16.

Receiver-operator-characteristic (ROC) curves from LOO and the single over-fit models are shown in Fig. 2. The area under the curve (AUC) for the single model was 1.0 (p-value 7.7e−19) and for the LOO models was 0.96 (p-value 5.6e−16). The Lasso modeling approach resulted in the identification of 55 metabolite peaks with all in common with important metabolite peaks listed in Table S3. The summary can be found in Supplementary Table S5).

**Figure 3:**
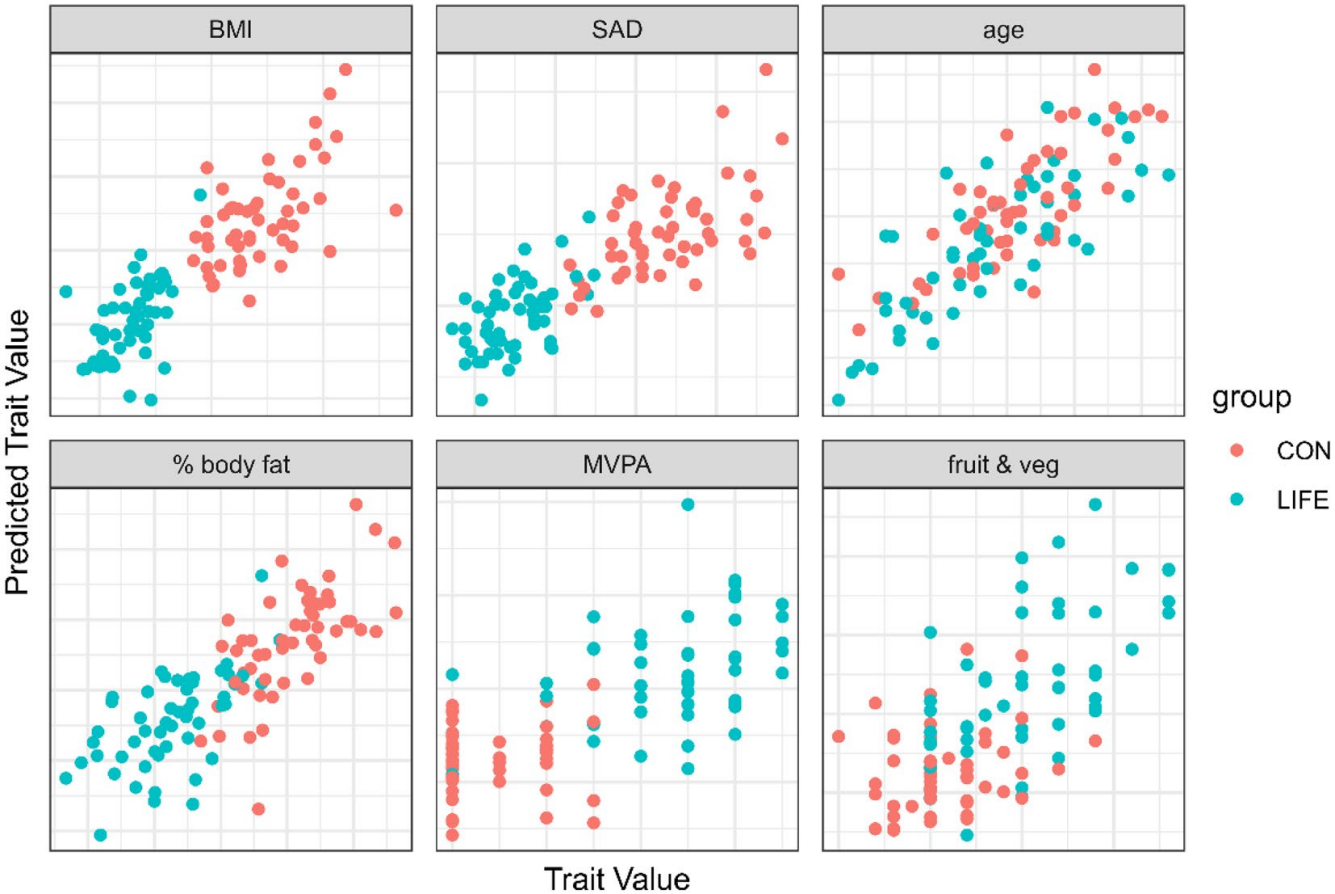
Leave-one-out cross validation (LOOCV) predictions using all metabolomics data for selected traits. Older age was strongly related to the metabolomics data with no differences between the LIFE and CON groups (r = 0.80, p = value = 5e−24). LIFE and CON group membership was strongly predicted using the plasma metabolomics data for three different body composition outcomes including BMI (r = 0.84, p-value = 3e−29), percent body fat (r = 0.80, p-value = 7e−24), and the sagittal abdominal diameter (SAD) (r = 0.82, p-value = 6e−27), and moderately predicted for the average number of daily servings of fruits and vegetables combined (r = 0.66, p-value = 3e−14), and the days per week for moderate-to-vigorous physical activity (MVPA) (r = 0.68, p-value = 4e−15).

LOO cross validation (LOOCV) predictions using all metabolomics data points are summarized in Fig. 3 for key lifestyle traits. Older age was strongly related to the metabolomics data with no differences between the LIFE and CON groups (r = 0.80, p = value = 5e−24). LIFE and CON group membership was strongly predicted using the plasma metabolomics data for three different body composition outcomes including BMI (r = 0.84, p-value = 3e−29), percent body fat (r = 0.80, p-value = 7e−24), and the sagittal abdominal diameter (SAD) (r = 0.82, p-value = 6e−27), and moderately predicted for the average number of daily servings of fruits and vegetables combined (r = 0.66, p-value = 3e−14), and the days per week for moderate-to-vigorous physical activity (MVPA) (r = 0.68, p-value = 4e−15).

### Pathway enrichment analysis

**Figure 4:**
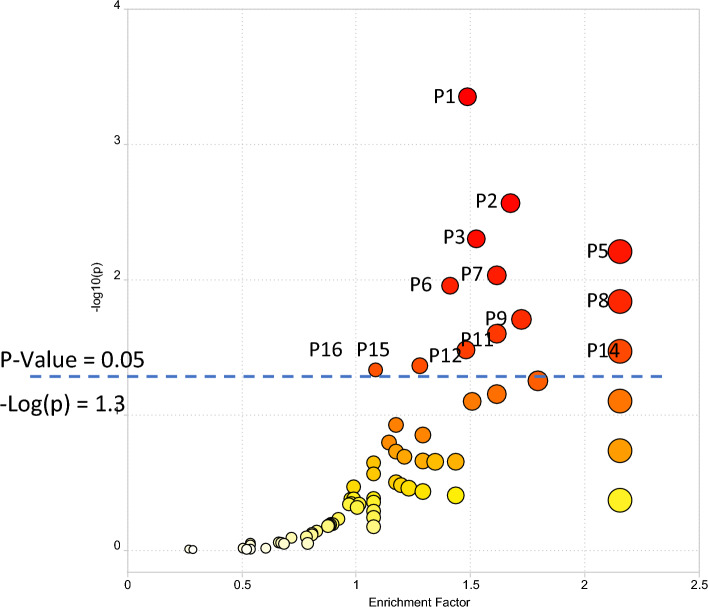
The enriched metabolic pathways for differentiating LIFE and CON groups using the Functional Analysis module in MetaboAnalyst. Top pathways significant (p < 0.05) in the analysis are annotated. A full list of enriched pathways is available in Supplementary Information Table S7. P1. Bile acid biosynthesis; P2. Histidine metabolism; P3. Lysine metabolism; P4. Heparan sulfate degradation; P5. Phytanic acid peroxisomal oxidation; P6. Glycosphingolipid metabolism; P7. Pyrimidine metabolism; P8. Chondroitin sulfate degradation; P9. N-Glycan degradation; P10. Aminosugars metabolism; P11. Beta-alanine metabolism; P12. Vitamin D3 (cholecalciferol) metabolism; P13. Glycosphingolipid biosynthesis—ganglioseries; P14. Glycosphingolipid biosynthesis—globoseries; P15. Butanoate metabolism; P16. Tyrosine metabolism. P4, P10, and P13 are not visible due to overlap.

Pathway enrichment was conducted using all 10,535 peaks in the dataset (with mass-to-charge ratio (m/z), retention time, the p-value, and fold change information as input) for comparison of LIFE versus CON phenotypic groups. The plot of pathway enrichment factor vs. –log10 (p) is shown in Fig. 4, and pathways deemed significant (p < 0.05) calculated by MetaboAnalyst are numbered (P1 to P16) in the figure. The top 16 enriched pathways (ranked by significance (p-value < 0.05) are shown in Table 3, and the complete list of the pathways are shown in the Supplementary Table S6 for the comparison of LIFE and CON groups. Signals associated with these enriched pathways, that were statistically significant between LIFE and CON groups, and that were identified or annotated using our in-house physical standards library are described in Table S7.

**Table 3:** The top 16 pathways (p ≤ 0.05) enriched and ranked in the Mummichog pathway analysis in MetaboAnalyst.

Pathway name	Pathway total^a^	Hits.total^b^	^c^Hits.sig^c^	FET^d^
Bile acid biosynthesis	82	50	42	0.000
Histidine metabolism	33	12	11	0.003
Lysine metabolism	52	15	15	0.005
Heparan sulfate degradation	34	3	3	0.006
Phytanic acid peroxisomal oxidation	34	5	5	0.006
Glycosphingolipid metabolism	67	15	13	0.009
Pyrimidine metabolism	70	19	16	0.011
Chondroitin sulfate degradation	37	3	3	0.015
N-Glycan Degradation	16	6	6	0.020
Aminosugars metabolism	69	8	8	0.025
Beta-Alanine metabolism	20	8	8	0.025
Vitamin D3 (cholecalciferol) metabolism	16	13	10	0.033
Glycosphingolipid biosynthesis—ganglioseries	62	4	4	0.034
Glycosphingolipid biosynthesis—globoseries	16	2	2	0.034
Butanoate metabolism	34	17	14	0.044
Tyrosine metabolism	160	63	48	0.047

^a^Pathway total indicates the overall number of metabolites that are included in a specific pathway.

^b^Hits.total indicates the number of measured signals that are matched (m/z error < 3 ppm) with the metabolites included in the pathway.

^c^Hits.sig indicates the number of matched signals that were significantly changed between phenotypic groups.

^d^FET is the right-tail p-value determined by the Fisher Exact Test for pathway enrichment.

## Discussion

This cross-sectional study focused on the metabotype profile associated with a healthy lifestyle. The LIFE and CON groups were of similar age, education, and sex distribution, but differed significantly in body composition and exercise and dietary patterns. The proteomics dataset previously published from this cross-sectional study showed strong group differences for 39 proteins supporting a lower innate immune activation signature and greater lipoprotein metabolism and HDL remodeling in the LIFE group^30^. In this analysis, untargeted metabolomics of more than 10,000 metabolite peaks revealed a distinct difference in the plasma metabolome between the LIFE and CON groups. Multivariate LOO modeling confirmed that group status (LIFE vs. CON) was strongly predicted by the metabolite signature and exceeded the prediction model from the proteomics data^30^. Numerous metabolites were identified/annotated that most significantly differentiated LIFE and CON groups. An enriched pathway analysis using Mummichog indicated group differences for 16 metabolic pathways highlighted by contrasts in bile acid and amino acid metabolism.

The reduced plasma bile acid signature in the LIFE vs. CON group is a novel and important finding from this cross-sectional study. Plasma hydroxycholesterol, a cholesterol precursor in primary bile acid metabolism, and more than 10 primary and secondary bile acids were significantly lower in the LIFE versus CON groups. Other studies indicate that plasma bile acid concentrations vary widely between individuals and that this variance is due to lifestyle, gut microbial, and genetic factors^50^. Normally enterohepatic circulation of bile acids is very efficient and only a small proportion of bile acids escape into the systemic circulation^50^. Circulating bile acids at normal low concentrations have regulatory functions and exert signaling functions in peripheral tissues and organs through specific nuclear receptors including the farnesoid X receptor (FXR) and the Takeda G protein-coupled receptor 1 (TGR5)^50,51^. Emerging data indicate that individuals with obesity and various diseases including type 2 diabetes mellitus have elevated plasma bile acid concentrations in the fasted state^48,49^. One study showed that even in young and relatively healthy adults, plasma bile acid levels were associated with cardiometabolic and inflammatory disease risk biomarkers^52^. A 14-week exercise and weight loss intervention study demonstrated that total fasting bile acids decreased by 30% accompanied by a 55% increase in serum levels of the rate-limiting enzyme cholesterol 7 alpha-hydroxylase (CYP7A1)^53^. Limited data suggest that aerobic capacity influences bile acid metabolism^54^ and that intake of dietary fiber and polyphenols from whole plant foods have a significant effect on the gut microbiome and bile acid metabolism and related signaling pathways^55,56^. Additional human systems biology-based studies using a variety of multiomics approaches will broaden current understandings regarding the specific and combined lifestyle relationships of body composition and dietary and exercise patterns on bile acid metabolism^50^.

LIFE versus CON group differences were found for seven of 20 standard amino acids, with higher histidine and lower glutamic acid, glutamine, β-alanine, phenylalanine, tyrosine, and proline. This LIFE-related amino acid signature was spread across seven different metabolic pathways including histidine, lysine, pyrimidine, amino sugars, β-alanine, tyrosine, and butanoate metabolism. Some aspects of this LIFE versus obese-CON-related amino acid signature have been reported by others, but the literature is far from consistent^18,26^. There is agreement that amino acid metabolism is extensively altered in various disease states and influenced by body composition and lifestyle habits^57^. In a cross-sectional study with obese and non-obese women serum amino acids including histidine, arginine, threonine, glycine, lysine, and serine were found to be significantly lower in obese women as compared to non-obese controls, similar to our results^58^. In our study, the most important LIFE versus CON contrast was for glutamic acid, an acidic, non-essential amino acid that is involved in numerous metabolic pathways. The plasma concentration of glutamic acid levels is inversely related to visceral adipose tissue and may be influenced by obesity-induced changes in the gut microbiota^59^. Pathway enrichment for LIFE versus CON identified the histidine metabolic pathway as most affected with higher levels of histidine, 4-imidazoleacetic acid, and l-formiminoglutamic acid and lower levels of glutamic acid in the LIFE group. Histidine is an essential amino acid and has been positively associated with insulin sensitivity, obesity, liver and kidney disease, and heart failure, and inversely related to inflammation and oxidative stress^58,60^. The gut microbiome appears to play a key role in regulating diet histidine bioavailability^61^. Plasma levels of branched chain amino acids (BCAAs) did not differ between LIFE and CON groups in contrast to other studies that have noted elevated plasma BCAA levels in obese groups^18,26^. The literature is mixed, however, regarding plasma BCAA levels and associations with adiposity, longevity, sarcopenia, and diabetes^62^.

5-hydroxylysine was increased and other lysine metabolites were decreased in LIFE versus CON groups. Limited data indicate that obesity may be related to enhanced lysine degradation via the saccharopine pathway^63^. Lysine is subjected to diverse enzyme-catalyzed post-translational modifications (PTMs), including methylation, acetylation, crotonylation, ubiquitination, and SUMOylation. Acetyllysine (or acetylated lysine) is an acetyl-derivative of the amino acid lysine. In proteins, the acetylation of lysine residues is an important mechanism of epigenetics. Free trimethyllysine (TML) is involved in the carnitine biosynthesis pathway, where it acts as the first intermediate in a series of four enzymatic reactions to generate l-carnitine^64^. TML is an important post-translationally modified amino acid with functions in carnitine biosynthesis and regulation of key epigenetic processes. The dataset from this cross-sectional study support lower levels of lysine degradation in the LIFE group, and the clinical significance of this finding remains to be determined. In contrast, pipecolic acid, an l-alpha amino acid metabolite product of lysine microbiome catabolism and a marker of dry bean intake^65^ was elevated in the LIFE group with a high VIP value of 2. There is increasing evidence that pipecolic acid is an important regulator of immunity in both plants and humans^66^.

Metabolites from the pyrimidine metabolism pathway including uracil, uridine, thymine, and 5-methylcytosine were higher in LIFE versus CON groups, with lower levels of glutamine, cytidine, cytosine, and pseudouridine. Uridine is an uracil nucleoside that is involved in a variety of biological functions including RNA and DNA biosynthesis, glucose and lipid metabolism, glycogen deposition, insulin sensitivity, energy homeostasis, protein and lipid glycosylation, extracellular matrix biosynthesis, and detoxification of xenobiotics^67^. Limited human data indicate that plasma uridine levels are inversely related to obesity^68^. In mice, uridine supplementation attenuates HFD-induced obesity and NAFLD^69^. A high uridine to pseudouridine ratio (as shown in the LIFE group) has been linked to a reduced risk for stroke^70^. Uridine decreases oxidative stress and inflammation in vitro and was linked to lower levels of aging indicators in mice^71^. Thus, alterations in plasma metabolites related to the pyrimidine pathway may serve as important and novel biomarkers of lifestyle habits and reduced disease risk.

Lifestyle habits had a positive influence on vitamin D3 (cholecalciferol) metabolism with higher plasma calcifediol (25(OH)D_3_) and calcitriol (1,25(OH)_2_D_3_) and lower 1,24,25-trihydroxyvitamin D3 (a 1,25(OH)_2_D_3_ catabolism metabolite) in the LIFE versus CON groups. A poor vitamin D status has been linked to obesity and numerous clinical conditions including the metabolic syndrome, type 2 diabetes mellitus, systemic inflammation, autoimmune disorders, and neurodegenerative diseases^70–75^. Underlying mechanisms for low vitamin D status in obese populations are unclear but may be related in part to reduced outdoor physical activity and volumetric dilution due to greater volumes of adipose tissue^73^.

The N-glycan degradation pathway analysis indicated reduced plasma levels in the LIFE group for mannose, galactose, N-acetylglucosamine, N-acetylneuraminic acid, and fucose. N-glycans (oligosaccharide-protein molecules) are basic components of cell membranes and secreted proteins and help regulate multiple physiological processes. In humans, N-glycosylation involves collections of mannose, galactose, fucose, and sialic acids including N-acetylneuraminic acid and N-acetylglucosamine. Sialic acids are acidic sugars typically located at the terminal positions of glycoproteins^76,77^. The amino sugars N-acetyl-d-mannosamine and N-acetyl-d-glucosamine (lower plasma levels in the LIFE group) are essential precursors of sialic acids. Plasma N-glycans and sialic acid levels are rather stable in healthy individuals over time but can be altered due to physiological, pathological, or lifestyle changes^76,77^. For example, elevated plasma levels of N-acetylneuraminic acid and N-acetylglucosamine have emerged as potential metabolic markers for inflammation, coronary artery disease progression, and a variety of other diseases^78,79^. Elevated plasma mannose has been reported in obese adults and is now considered a biomarker for future risk of several chronic diseases^18,80,81^. Increased l-fucose in serum and urine is a potential biomarker for cancer, diabetes, cardiovascular disease, cirrhosis, alcoholic liver disease and gastric ulcers^82,83^. The markedly lower plasma levels of N-glycan degradation metabolites in the LIFE group supports the interpretation of reduced chronic disease risk due to positive lifestyle habits. N-acetylglucosamine when polymerized with glucuronic acid forms heparin sulfate and is distributed throughout connective, neural, and epithelial tissues. Lower levels of plasma N-acetylglucosamine support the pathway analysis finding of a lowered degradation of heparan sulfate in the LIFE group^84^.

Due to limitations in the curation of metabolites in the library of the Mummichog analysis modules, metabolites including those related to gut microbiome catabolism of food substrates and environmental contaminants were not included in the pathway analysis. Several gut microbiome metabolites reflecting a higher intake of plant-based foods and enhanced gut microbiome alpha diversity were elevated in the LIFE versus CON group including hippuric acid, cinnamoylglycine, cinnamic acids, 3,4-dimethoxyphenylacetic acid, 3-phenylpropanoic acid, and 2-phenylpropionate. An elevated gut microbial metabolite signature in adults with higher lifestyle scores has been reported previously^85^. Citric acid cycle metabolites generated from the butanoate metabolism pathway differed between LIFE and CON groups, with higher levels of succinic acid. The butanoate metabolism pathway involves short chain fatty acids (SCFA) produced by bacterial fermentation of undigested carbohydrates (including dietary fiber) and proteins. SCFAs are precursors for numerous metabolites including succinic acid that helps regulate cellular nutrient metabolism and white adipose tissue deposition, muscle fiber remodeling during recovery from exercise, and immune system function^86^. Plasma levels of two disaccharides, lactose and sucrose, are indicators of a leaky gut syndrome and were lower in the LIFE versus CON group.

Plasma levels of numerous environmental contaminants were lower in the LIFE versus CON groups including propham (a potato herbicide), fenoxycarb (carbamate-based insecticide metabolite), monocyclohexyl phthalate and (5-Carboxy-2-ethylpentyl)phthalate (plasticizer metabolites), prometon (an herbicide), 3-hydroxycarbofuran (a pesticide carbofuran metabolite), furalaxyl and propamocarb free base (fungicides), and 9-hydroxyfluorene (insecticide and algaecide). Two other cross-sectional studies showed lower levels of blood persistent organochlorine pesticides (POPs) in lean or physically active compared to obese or sedentary adults^87,88^. Dietary, lifestyle, and environmental exposures are still being investigated, but some of the environmental contaminants identified in this study tend to accumulate in the fatty tissues of commonly consumed livestock. Thus, a higher intake of red meat fat in the CON group may have increased the body-exposure burden of environmental contaminants^89^.

Pathway enrichment identified LIFE versus CON differences in the glycosphingolipid biosynthesis and metabolism pathway, with higher levels of the key metabolite phosphorylcholine. Glycosphingolipids (GSLs) are a specialized class of membrane lipids that support various cellular functions. Phosphorylcholine (PC) is the hydrophilic polar head group of some phospholipids and is a component of the platelet-activating factor and the phospholipids phosphatidylcholine and sphingomyelin^90^. Non-pathogenic antibodies against PC are naturally occurring and present in healthy adults. About 5–10% of circulating immunoglobulin M (IgM) consists of IgM anti-PC. IgM anti-PC is negatively associated with several chronic inflammatory conditions, including atherosclerosis, CVD, rheumatic diseases and chronic kidney disease (CKD)^90^.

Other metabolites of importance that were elevated in the LIFE group included beneficial fatty acids such as γ-linolenic acid, docosahexaenoic acid (DHA) and eicosatetraenoic acid (EPA). Lower levels of beneficial fatty acids have been reported in obese populations^18^. The reduced form of glutathione was significantly elevated in the LIFE group and is an indicator of reduced oxidative stress^91^. Tryptamine, 2-hydroxyethyl)indole, and serotonin are gut microbial catabolites of tryptophan and were elevated in the LIFE group. These metabolites play roles in the gut-brain axis, immune surveillance, and inflammation regulation^92^. Two other gut microbial catabolites of tryptophan were decreased in the LIFE group including indole-3-methyl acetate and indole-3-propionic acid. Plasma betaine and lutein levels were higher in the LIFE group. Betaine is a methyl donor, regulates osmotic pressure, has positive effects on intestinal and kidney health, and exerts anti-inflammatory and anti-oxidative effects^93^. Lutein is a common carotenoid in plant foods. Several metabolites related to pain relief medications were elevated in the CON group, with higher levels of acetaminophen higher in the LIFE group. Plasma nicotine and cotinine were higher in the LIFE group and may indicate a higher prevalence of vaping.

## Conclusions

The plasma metabolome reflects the collective influence of multiple lifestyle habits, genotype, clinical stressors, the gut microbiota, and other factors^94^. This cross-sectional study investigated differences in the plasma metabolome in two groups of adults that varied widely in body composition and dietary and physical activity patterns. The results using an extensive untargeted UPLC-HRMS analysis with more than 10,000 metabolite peaks identified/annotated numerous metabolites and 16 metabolic pathways that differentiated LIFE and CON groups. A novel metabolite signature of positive lifestyle habits emerged from this analysis highlighted by lower plasma levels of numerous bile acids and an amino acid profile consistent with a reduced risk for chronic disease. This analysis also supported an elevated vitamin D status in the LIFE group, higher levels of beneficial fatty acids and gut microbiome catabolism metabolites from plant substrates, and reduced levels of N-glycan degradation metabolites and environmental contaminants. The LOOCV analysis supported the strong effect that body composition had on the plasma metabolome, with moderate effects of MVPA and fruit and vegetable intake. We propose that low-cost anthropometrics measurements could be combined with important metabolites from this analysis as precision nutrition indicators of a healthy versus unhealthy lifestyle. These metabolites could include lower plasma levels of glutamic acid, total bile acids, N-acetylneuraminic acid, and mannose, and higher levels of histidine, pipecolic acid, L-glutathione (reduced), succinic acid, γ-linolenic, DHA, EPA, hippuric acid, calcitriol, phosphorylcholine, uridine, 5-hydroxylysine, betaine, and lutein.

### Supplementary Information

Supplementary Information.

Supplementary Table S1.

Supplementary Table S2.

Supplementary Table S3.

Supplementary Table S4.

Supplementary Table S5.

Supplementary Table S6.

Supplementary Table S7.

## Data Availability

The datasets used and/or analyzed during the current study are available from the corresponding author on reasonable request. The normalized and raw metabolomics datasets generated during the current study are available at the NIH Common Fund's National Metabolomics Data Repository (NMDR), https://www.metabolomicsworkbench.org (Project ID: PR001932). The metabolomics data package is embargoed to the public for a year to accommodate publishing results. During the embargo period, anyone with this link can access the data: http://dev.metabolomicsworkbench.org:22222/data/DRCCMetadata.php?Mode=Study&StudyID=ST003110&Access=TreQ7245.
