# Supplementary material for: Untargeted metabolomics reveal signatures of a healthy lifestyle
Source: Sci Rep. 2024 Jun 13;14:13630. doi: 10.1038/s41598-024-64561-z (PMC11176323; doi:10.1038/s41598-024-64561-z)
Supplement: Supplementary file 1 — Supplementary Information. [file 41598_2024_64561_MOESM1_ESM.docx]

**Title: Untargeted Metabolomics Reveal Signatures of a Healthy Lifestyle**

**Supplementary Methods Material**

Wimal Pathmasiri^1, 2, #^, Blake R. Rushing^1, 2, #^, Susan McRitchie^2^, Mansi Choudhari^2^, Xiuxia Du^3^, Alexsandr Smirnov^3^, Matteo Pelleigrini^4^, Michael J. Thompson^4^, Camila A. Sakaguchi^5^, David C. Nieman^5*^ & Susan J. Sumner^1, 2, *^

^1^Department of Nutrition, University of North Carolina at Chapel Hill, Chapel Hill, NC 27599, USA

^2^Nutrition Research Institute, University of North Carolina at Chapel Hill, Kannapolis, NC 28081, USA

^3^ College of Computing and Informatics, UNC Charlotte, Kannapolis, NC, 28081, USA

^4^ Department of Molecular, Cell, and Developmental Biology, University of California Los Angeles, Los Angeles, CA, USA.

^5^Human Performance Laboratory, Department of Biology, Appalachian State University, North Carolina Research Campus, Kannapolis, NC 28081

# Co-first Authors contributed equally

*Correspondence for Metabolomics

Susan Sumner, PhD

[susan_sumner@unc.edu](mailto:susan_sumner@unc.edu)

*Correspondence for Human Cohort Study:

David Nieman, DrPH, FACSM

[niemandc@appstate.edu](mailto:niemandc@appstate.edu)

**Keywords:** metabolomics, UHPLC-high resolution mass spectrometry, LCMS, lifestyle, obesity

**Running Title:** Healthy Lifestyle Metabotypes

| 1 | Sample preparation, data acquisition, data preprocessing, and metabolite identification and annotation for untargeted metabolomics via UPLC high resolution mass spectrometry. |  |
| --- | --- | --- |
| 2 | Supplemental Table S1.xlsx |  |
| 3 | Supplemental Table S2.xlsx |  |
| 4 | Supplemental Table S3.xlsx |  |
| 7 | Supplemental Table S4.xlsx |  |
| 6 | Supplemental Table S5.xlsx |  |
| 7 | Supplemental Table S6.xlsx |  |
| 8 | Supplemental Table S7.xlsx |  |
| 9 | Supplemental Figure S1 (this document) |  |

**Checklist for Supplementary Material**

**Supplementary Material**

1. **Sample preparation, data acquisition, data preprocessing and metabolite identification and annotation for untargeted metabolomics via UHPLC-high resolution mass spectrometry (UHPLC-HRMS)**.

Sample preparation and Data Acquisition: Plasma samples were prepared according to the published methods^1-3^. In brief, 50 µL of plasma sample was mixed with 400 µL methanol containing 500 ng/ml L-tryptophan-d5, and vortexed at 5,000 rpm for 2 min. Samples were incubated for 10 minutes at 4°C and centrifuged at 16, 000 rcf for 5 min at 4°C. The supernatant (350 µL) was dried and reconstituted with 100 µl water-methanol (95:5, v/v), vortexed for 10 min at 5000 rpm, and then centrifuged at 16,000 rcf for 10 min at 4°C. The supernatant was transferred to pre-labeled autosampler vials. Quality control samples (QCSP) were prepared by pooling 10 µL plasma from each of the study samples. A 50 µL aliquot of blank (LCMS grade water), NIST plasma SRM 1950, and QCSP sample was processed identically to the study samples. The study samples were randomized with interspersed QCSP, blanks, and NIST plasma before data acquisition.

Metabolomics data was acquired on a Vanquish UHPLC systems coupled with a Q Exactive™ HF-X Hybrid Quadrupole-Orbitrap™ Mass Spectrometer (Thermo Fisher Scientific, San Jose, CA). A 5 µL volume was injected onto the UHPLC column for untargeted analysis. Metabolites were separated via an HSS T3 C18 column (2.1 x 100 mm, 1.7 µm, Waters Corporation, Milford, MA) at 50 °C with binary mobile phases, which are water (A) and methanol (B), each containing 0.1% formic acid (v/v). The UHPLC linear gradient started from 2% B, and increased to 100% B in 16 min, then held for 4 min, with a flow rate at 0.4 ml/min. The untargeted data was collected from 70 to 1050 m/z in positive mode, under the data dependent acquisition (DDA) mode. This study was conducted in the positive mode only and could have missed peaks that may have arisen only in the negative mode. In addition, this study only used UHPLC-HRMS and may have missed peaks that would have been detected by our other technologies (e.g., NMR, GC-MS, CE-MS, cytokine arrays).

Metabolomics Data Preprocessing: The untargeted UHPLC-HRMS data was processed by Progenesis QI (version 2.1, Waters Corporation) for peak picking, alignment, and normalization. The data matrix was normalized^4, 5^ in Progenesis QI using the “normalize to total intensity” feature^6^. Principal Component Analysis was used to visualize the clustering of QCSP samples and NIST plasma reference material (**Supplementary Figure S1)**. The coefficient of variation (CV) of Tryptophan-d5 was 7.2%.

Metabolite Identification and Annotation: Peaks detected by UPLC-HR-MS were identified or annotated through matching to an in-house physical standards library generated by acquiring data for over 2400 compounds under identical conditions to the study samples, as well as to public databases (including HMDB, METLIN, and NIST) using ADAP-KDB software^7^. Identifications and annotations used available data for retention time (RT), exact mass (MS), MS/MS fragmentation pattern, and isotopic pattern. An evidence-based ontology level (OL) was given for the metabolite identification/ annotations. Signals/metabolites reported in the results and discussion section that matched to the in-house experimental standards library by (a) RT, MS, and MS/MS are labeled as OL1, or (b) by RT and MS are labeled OL2a. An OL2b label was provided for signals that matched by MS and MS/MS to the in-house library, that were outside the retention time tolerance (±0.5 min) for the standards run under identical conditions. Note that D, L, and structural isomers are not necessarily differentiated on the untargeted platform. Signals matched to public databases are labeled as PDa (MS and experimental MS/MS), PDc (mass match and isotope similarity), or PDd (mass match) are also provided in supplemental material.

**Supplementary tables**

1. **Supplemental Table S1**. Metabolites identified or annotated using the in-house physical standards library using ADAP-KDB.
2. **Supplemental Table S2**. Metabolite annotations made by matching signals to public databases (NIST, HMDB, ToxCast) using ADAP-KDB.
3. **Supplemental Table S3**. Metabolites meeting the criteria of VIP ≥1 or p < 0.10, or absolute value of fold change ≥ 2.
4. **Supplemental Table S4**. Important metabolite peaks (VIP ≥1 or p < 0.10, or absolute value of fold change ≥ 2) matched to metabolites in the in-house physical standards library using ADAP-KDB.
5. **Supplemental Table S5**. Metabolites identified in the Lasso modeling and metabolomics approaches.
6. **Supplemental Table S6**. Complete list of enriched pathways.
7. **Supplemental Table S7**. Significantly enriched metabolic pathways (Table 3 and Figure 5) between LIFE and CON groups and annotated/ identified metabolites (using in-house physical standards library) that can be assigned to these pathways. Increased=Level of metabolite is increased in LIFE group compared to CON group. The ontology level and p-value of the annotated peak is depicted in parentheses.

**Supplementary Figure**

**
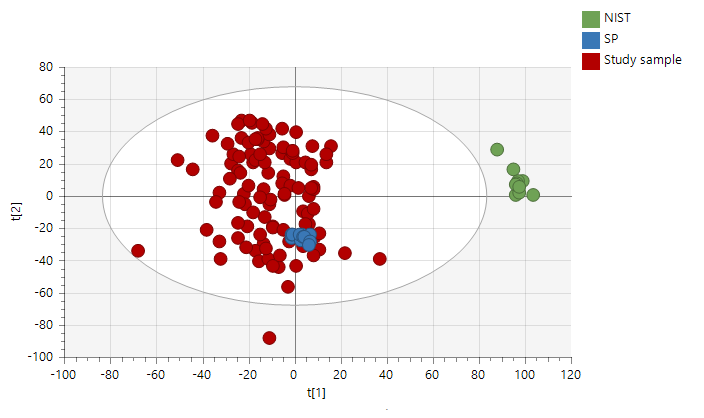
**

1. **Supplementary Figure S1.** Scores plot of PCA showing tight clustering of quality control study pool (QCSP) samples (blue) and NIST reference plasma (green) samples (red=Study Samples).

**References**

1 Li, Y. Y. *et al.* Untargeted Metabolomics: Biochemical Perturbations in Golestan Cohort Study Opium Users Inform Intervention Strategies. *Front Nutr* **7**, 584585, doi:10.3389/fnut.2020.584585 (2020).

2 Ghanbari, R. *et al.* Metabolomics reveals biomarkers of opioid use disorder. *Transl Psychiatry* **11**, 103, doi:10.1038/s41398-021-01228-7 (2021).

3 Lynch, D. H. *et al.* Baseline Serum Biomarkers Predict Response to a Weight Loss Intervention in Older Adults with Obesity: A Pilot Study. *Metabolites* **13**, doi:10.3390/metabo13070853 (2023).

4 Sun J, Xia Y. Pretreating and normalizing metabolomics data for statistical analysis. Genes Dis. 2023 Jul 7;11(3):100979. doi: 10.1016/j.gendis.2023.04.018. PMID: 38299197; PMCID: PMC10827599.

5 Chan EC, Pasikanti KK, Nicholson JK. Global urinary metabolic profiling procedures using gas

chromatography-mass spectrometry. Nat Protoc. 2011 Sep 8;6(10):1483-99. doi:

10.1038/nprot.2011.375. PMID: 21959233.

6 Valikangas, T., Suomi, T. & Elo, L. L. A systematic evaluation of normalization methods in quantitative label-free proteomics. *Brief Bioinform* **19**, 1-11, doi:10.1093/bib/bbw095 (2018).

7 Smirnov, A., Liao, Y., Fahy, E., Subramaniam, S. & Du, X. ADAP-KDB: A Spectral Knowledgebase for Tracking and Prioritizing Unknown GC-MS Spectra in the NIH's Metabolomics Data Repository. *Anal Chem* **93**, 12213-12220, doi:10.1021/acs.analchem.1c00355 (2021).
